# Supplementary material for: Phylogeography and Evolutionary Dynamics of Tobacco Curly Shoot Virus
Source: Viruses. 2024 Nov 28;16(12):1850. doi: 10.3390/v16121850 (PMC11680240; doi:10.3390/v16121850)
Supplement: Supplementary file 1 [file viruses-16-01850-s001.zip › Table S2.pdf]

Table S2 Marginal likelihoods of different combinations of clock model and tree prior

Model of rate variation	Tree prior	Log marginal likelihood	
		Path Sampling	Step-stone sampling
**Strict clock**	**Bayesian skyline**	**-3677.988**	**-3677.981**
Strict clock	Constant size	-3682.209	-3681.160
Strict clock	Exponential growth	-3686.334	-3684.795
Uncorrelated lognormal relaxed clock	Bayesian skyline	-3679.746	-3678.473
Uncorrelated lognormal relaxed clock	Constant size	-3683.713	-3685.628
Uncorrelated lognormal relaxed clock	Exponential growth	-3686.751	-3687.979

The best-fitting tree prior and molecular clock model are indicated in bold font.
