# Supplementary figures and images for: Phylogeography and Evolutionary Dynamics of Tobacco Curly Shoot Virus
Source: Viruses. 2024 Nov 28;16(12):1850. doi: 10.3390/v16121850 (PMC11680240; doi:10.3390/v16121850)

a.

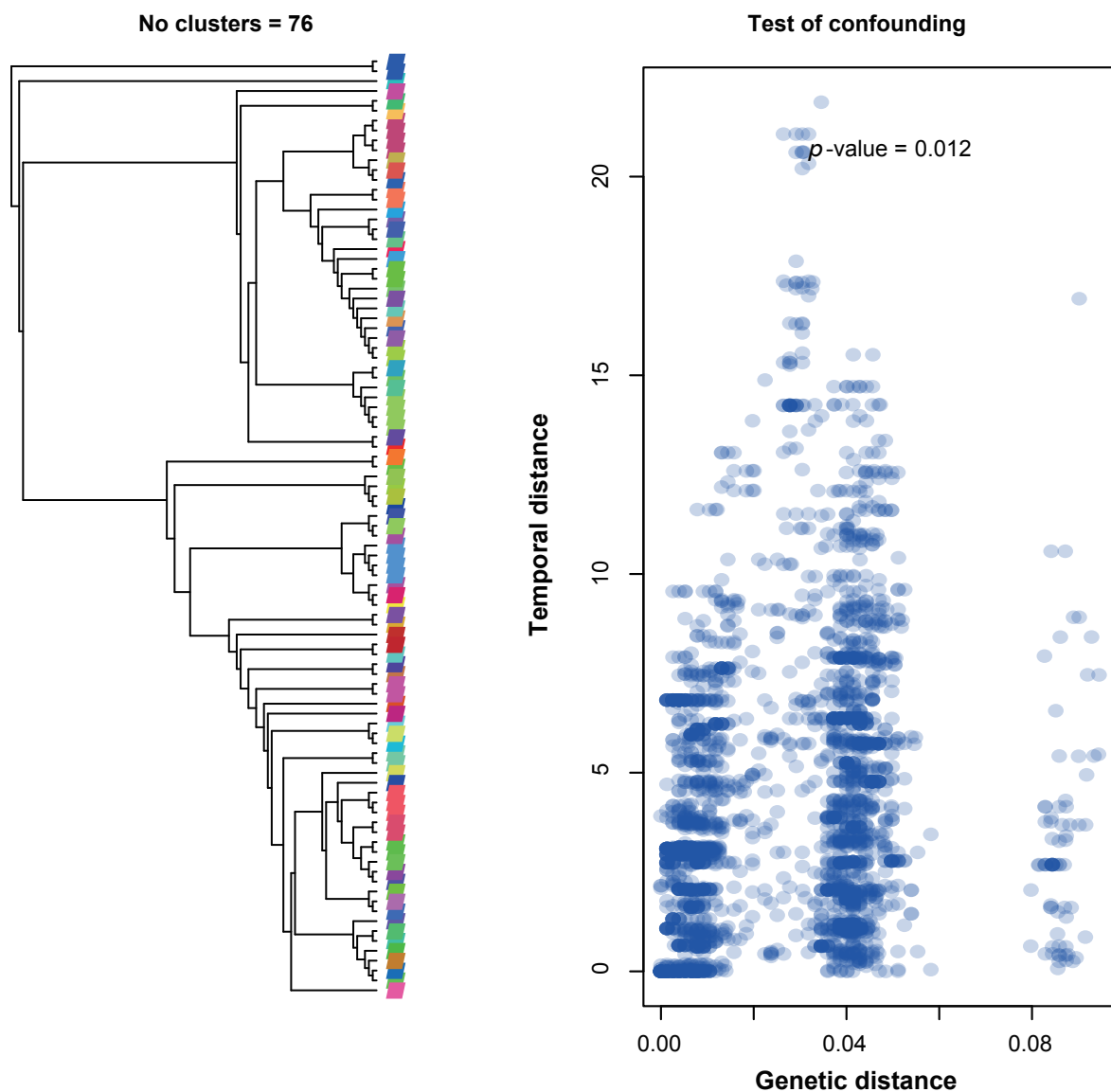

b.

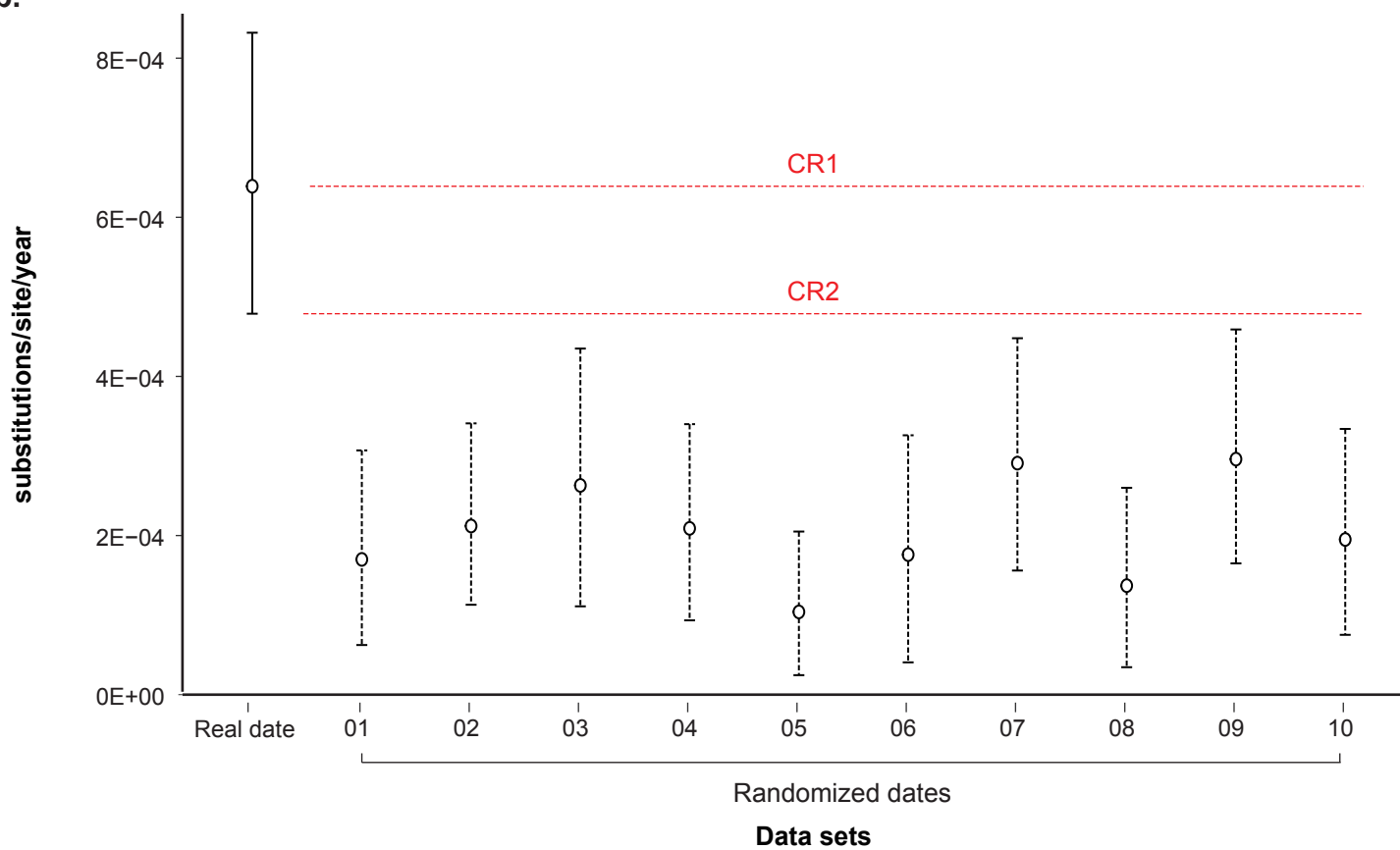

Supplement: Supplementary file 1 [file viruses-16-01850-s001.zip › Figure_S1.pdf]

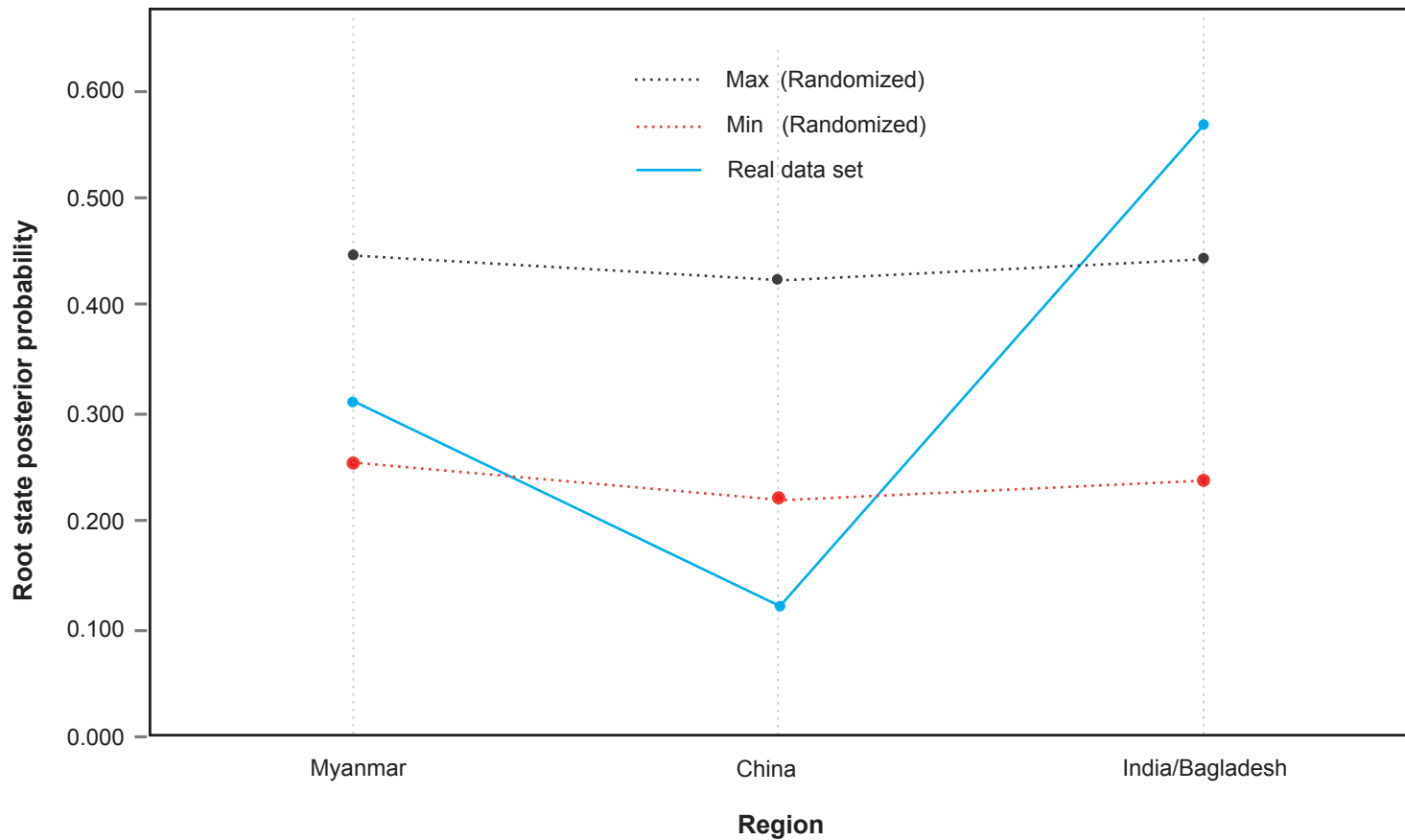

Supplement: Supplementary file 1 [file viruses-16-01850-s001.zip › Figure_S2.pdf]

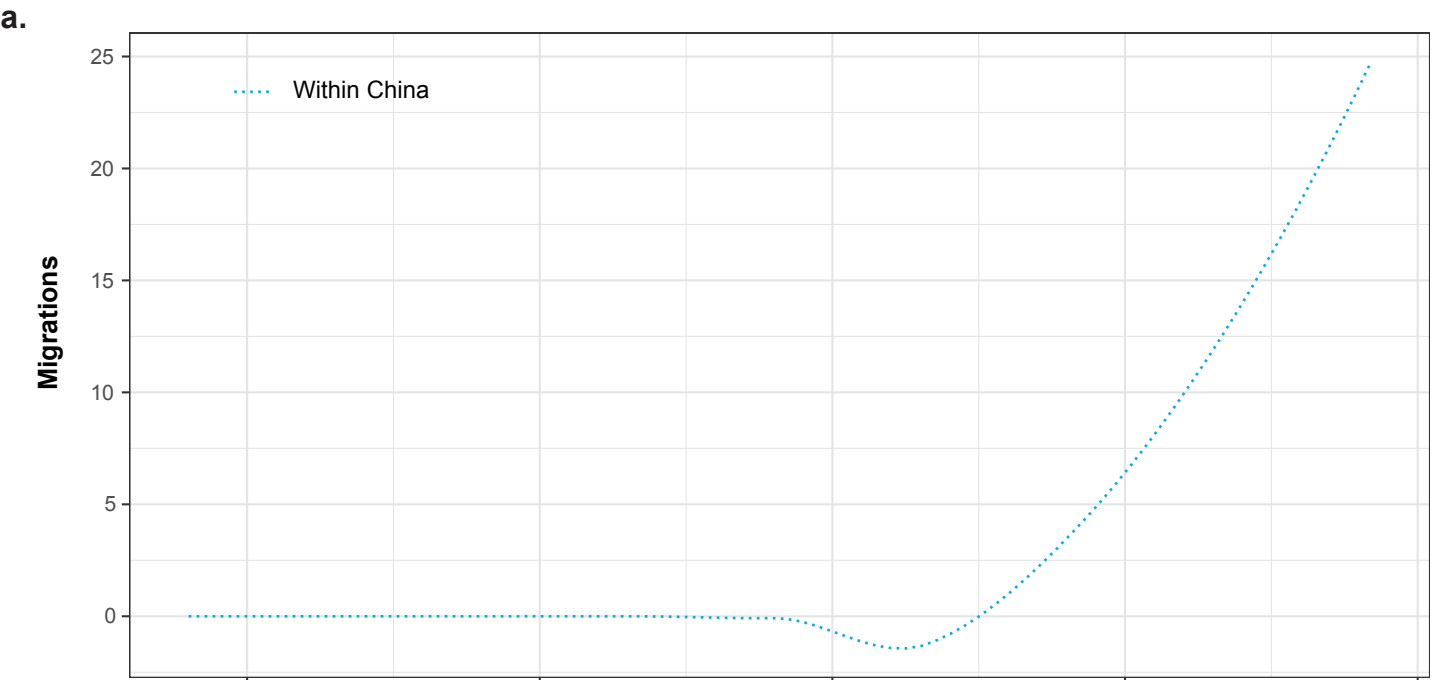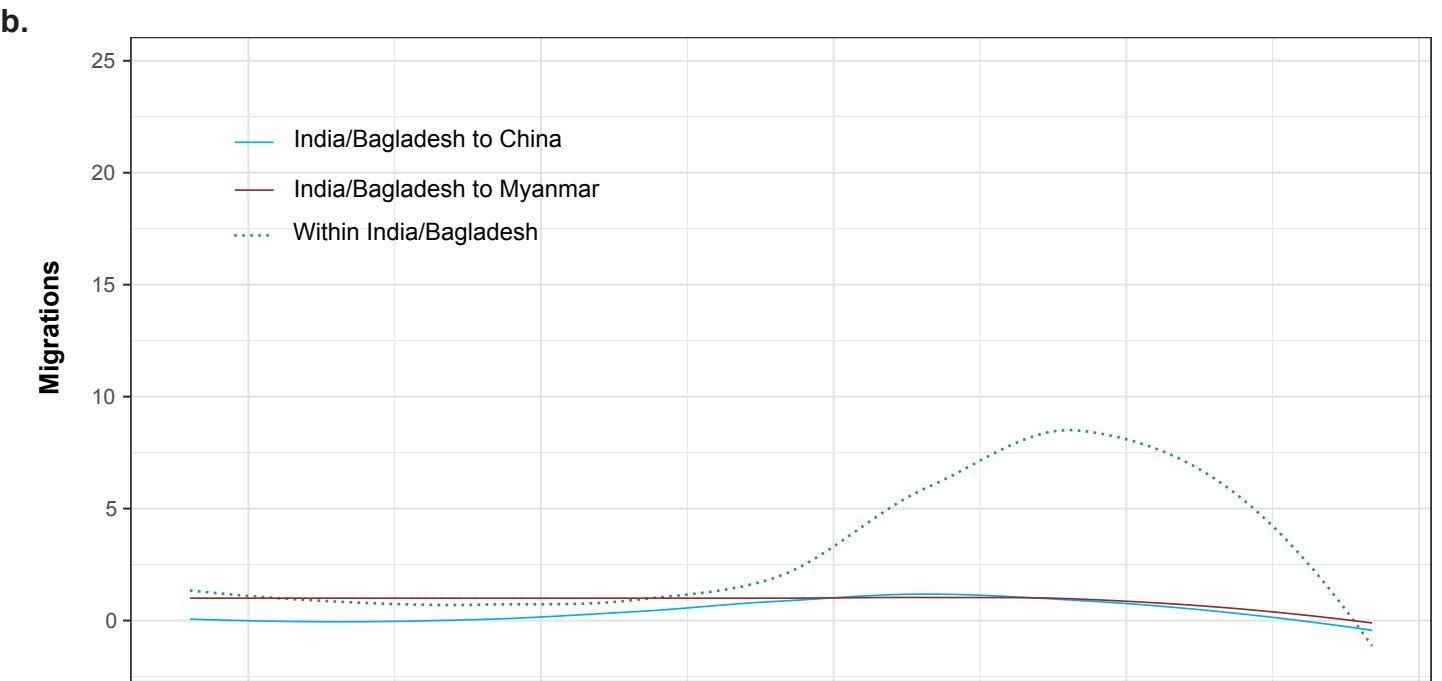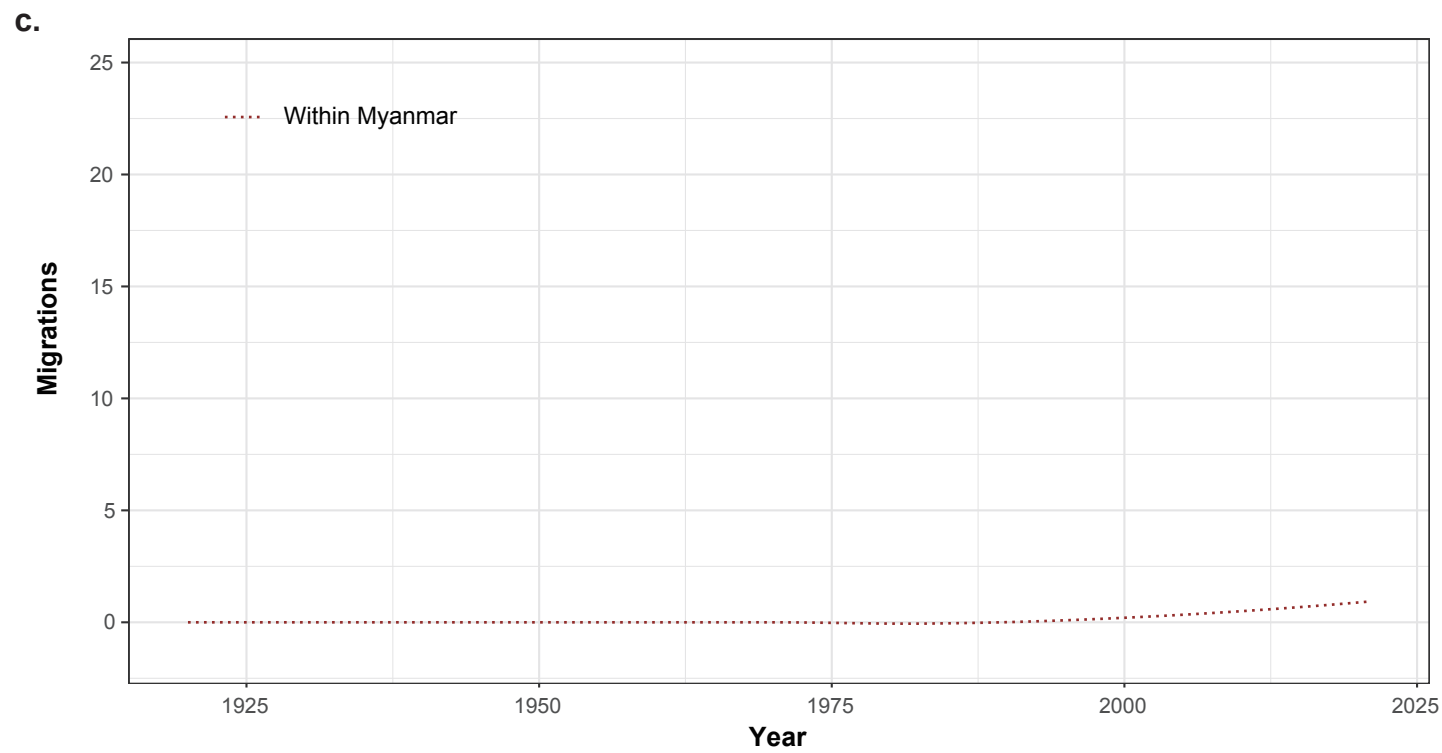

Supplement: Supplementary file 1 [file viruses-16-01850-s001.zip › Figure_S3.pdf]
